# Plasma biomarker profiles in autosomal dominant Alzheimer’s disease

**DOI:** 10.1093/brain/awac399

**Published:** 2023-01-11

**Authors:** Charlotte Johansson, Steinunn Thordardottir, José Laffita-Mesa, Elena Rodriguez-Vieitez, Henrik Zetterberg, Kaj Blennow, Caroline Graff

**Affiliations:** Division of Neurogeriatrics, Department of Neurobiology, Care Sciences and Society, Karolinska Institutet, Solna, Sweden; Theme Inflammation and Aging, Karolinska University Hospital, Stockholm, Sweden; Memory Clinic, Department of Geriatrics, Landspitali University Hospital, Reykjavik, Iceland; Division of Neurogeriatrics, Department of Neurobiology, Care Sciences and Society, Karolinska Institutet, Solna, Sweden; Division of Neurogeriatrics, Department of Neurobiology, Care Sciences and Society, Karolinska Institutet, Solna, Sweden; Division of Clinical Geriatrics, Department of Neurobiology, Care Sciences and Society, Karolinska Institutet, Stockholm, Sweden; Clinical Neurochemistry Laboratory, Sahlgrenska University Hospital, Mölndal, Sweden; Department of Psychiatry and Neurochemistry, Institute of Neuroscience and Physiology, the Sahlgrenska Academy, University of Gothenburg, Mölndal, Sweden; Department of Neurodegenerative Disease, UCL Institute of Neurology, London, UK; UK Dementia Research Institute at UCL, London, UK; Clinical Neurochemistry Laboratory, Sahlgrenska University Hospital, Mölndal, Sweden; Department of Psychiatry and Neurochemistry, Institute of Neuroscience and Physiology, the Sahlgrenska Academy, University of Gothenburg, Mölndal, Sweden; Division of Neurogeriatrics, Department of Neurobiology, Care Sciences and Society, Karolinska Institutet, Solna, Sweden; Theme Inflammation and Aging, Karolinska University Hospital, Stockholm, Sweden

**Keywords:** autosomal dominant Alzheimer's disease, plasma biomarker, tau, glial fibrillary acidic protein, neurofilament light chain

## Abstract

Emerging plasma biomarkers of Alzheimer's disease might be non-invasive tools to trace early Alzheimer's disease-related abnormalities such as the accumulation of amyloid-beta peptides, neurofibrillary tau tangles, glial activation and neurodegeneration. It is, however, unclear which pathological processes in the CNS can be adequately detected by peripheral measurements and whether plasma biomarkers are equally applicable in both clinical and preclinical phases. Here we aimed to explore the timing and performance of plasma biomarkers in mutation carriers compared to non-carriers in autosomal dominant Alzheimer's disease.

Samples (*n* = 164) from mutation carriers (*n* = 33) and non-carriers (*n* = 42) in a Swedish cohort of autosomal dominant Alzheimer's disease (*APP* p.KM670/671NL, *APP* p.E693G and *PSEN1* p.H163Y) were included in explorative longitudinal analyses. Plasma phosphorylated tau (P-tau181), total tau (T-tau), neurofilament light chain (NfL) and glial fibrillary acidic protein (GFAP) concentrations were measured with a single-molecule array method as previously described. Plasma biomarkers were additionally correlated to Alzheimer's disease core biomarkers in the CSF.

Results from the longitudinal analyses confirmed that plasma P-tau181, NfL and GFAP concentrations were higher in mutation carriers compared to non-carriers. This change was observed in the presymptomatic phase and detectable first as an increase in GFAP approximately 10 years before estimated symptom onset, followed by increased levels of P-tau181 and NfL closer to expected onset. Plasma P-tau181 levels were correlated to levels of P-tau181 and T-tau in the CSF.

Altogether, plasma P-tau181, GFAP and NfL seem to be feasible biomarkers to detect different Alzheimer's disease-related pathologies already in presymptomatic individuals. Interestingly, changes in plasma GFAP concentrations were detected prior to P-tau181 and NfL. Our results suggest that plasma GFAP might reflect Alzheimer's disease pathology upstream to accumulation of tangles and neurodegeneration. The implications of these findings need additional validation, in particular because of the limited sample size.

## Teaser

Johansson *et al.* describe plasma biomarkers in a longitudinal Swedish cohort of patients with monogenic Alzheimer’s disease. Plasma GFAP began to increase 10 years before symptom onset, prior to P-tau181 and NfL, suggesting that GFAP is mirroring Alzheimer’s disease pathology upstream of tau phosphorylation and neurodegeneration.

## Introduction

Autosomal dominant Alzheimer's disease (ADAD) is caused by pathogenic mutations in the amyloid precursor protein (*APP*), presenilin 1 (*PSEN1*) or presenilin 2 (*PSEN2*) genes. ADAD shares the neuropathological hallmarks of Alzheimer's disease, i.e. neuritic plaques and neurofibrillary tangles, composed of amyloid-beta (Aβ) peptides and hyperphosphorylated tau, respectively.^1^ The order of ADAD pathologies, as measured by biomarker abnormalities in presymptomatic and symptomatic phases of the disease continuum, are reported to largely conform to those in sporadic Alzheimer's disease^2,3^ and, also, align to the A/T/N (Aβ, tau and neurodegeneration) classification.^4,5^ These similarities and the deterministic and predictable onset of symptoms in ADAD mutation carriers (MC) have made ADAD an important model for sporadic Alzheimer's disease in general.

Validated blood-based biomarkers at low cost would be broadly useful for clinical practice and research settings. Alzheimer's disease core biomarkers of tau pathology and neurodegeneration, while changing downstream to Aβ pathology and closer to symptom onset, could be applicable to monitor disease progression and disease activity, which is likely required in upcoming clinical trials. There is also growing evidence that glial activation and neuroinflammation have a role from early stages of Alzheimer's disease, and fluid biomarkers of glial activation are now emerging.^6^ Previously, plasma measurements of tau phosphorylated at threonine 181 (P-tau181) have shown good accuracy in distinguishing between Aβ-positive and -negative individuals, as measured by analysis of CSF and PET, between sporadic Alzheimer's disease and other neurodegenerative diseases and, also, good accuracy in predicting cognitive decline.^7–9^ CSF glial fibrillary acidic protein (GFAP), a marker of astrogliosis, increases in several neurodegenerative disorders, while serum GFAP has shown better specificity for Alzheimer's disease.^10^ Neurofilament light chain (NfL) and total tau (T-tau) are more non-specific biomarkers of neurodegeneration.^4^ NfL is enriched in large-calibre neuronal axons and the concentration of NfL in CSF increases in multiple neurodegenerative disorders, is not specific for Alzheimer's disease and has rather been suggested as a biomarker of disease severity.^11^ Plasma NfL has previously been shown to outperform plasma T-tau both in distinguishing between controls versus sporadic Alzheimer's disease and in predicting cognitive decline.^12^ Several of these biomarkers have shown promising results in the symptomatic phase but are incompletely studied in the presymptomatic or preclinical phase and questions remain in regard to timing, reproducibility, generalizability and correlation to pathology in the CNS.

We aimed to evaluate the longitudinal trajectories of plasma biomarkers in presymptomatic (PMC) and symptomatic mutation carriers (SMC) in a Swedish ADAD cohort. Ultrasensitive biochemical measures of plasma P-tau181, T-tau, NfL and GFAP were investigated at baseline and in longitudinal analyses. Further, we investigated the association of these novel plasma biomarker concentrations with biomarkers of P-tau181, T-tau and various Aβ fragments in the CSF.

## Materials and methods

### Study design and participants

The participants were adult relatives at risk of ADAD in a Swedish longitudinal prospective study (the Swedish familial Alzheimer's disease study) ongoing since the 1990s, and came from two *APP* (*APPswe*, p.KM670/671NL and *APParc*, p.E693G) families and one *PSEN1* (*PSEN1* p.H163Y) family. All participants contributed with either blood samples and clinical data or participated in an extensive study protocol including neuroimaging (3 T MRI), EEG, cognitive assessment, CSF, skin and blood sampling, at varying follow-up intervals. Mutation status was unknown to participants and study personnel, unless a clinical presymptomatic genetic test had been requested by the participant. Family members included in the plasma and CSF biomarker analyses were classified as either MC (participants with the disease-causing mutation) or non-carriers (NC, participants without the mutation). The non-carriers from all three families were grouped together and used as a reference group in the statistical analyses (controls). The mean age at symptom onset was 54 ± 5 years (mean ± SD) in *APPswe* MC (based on 24 affected individuals), 56 ± 4 years in *APParc* (based on 15 affected individuals) and 52 ± 6 years in the *PSEN1* p.H163Y family (based on 12 affected individuals).

Sampling was performed during the years 1994 to 2018. All plasma and CSF biomarkers were analysed at the Clinical Neurochemistry Laboratory at the Sahlgrenska University Hospital, Mölndal Sweden.

Informed written consent was obtained for inclusion of all participants. The study was conducted in accordance with the Helsinki declaration and approved by the Regional Ethical Review Board in Stockholm, Sweden.

#### Estimated years to symptom onset

In ADAD, the mean age at onset in every family (for each mutation) can be used to estimate the expected age at onset in at risk individuals, which in turn can be used for comparisons between kindreds.^13^ Here, the mean age at onset was calculated per mutation and this ‘mean age’ was subtracted from the actual age at sampling to generate the variable ‘estimated years to symptom onset’ (EYO) for each sampling occasion in both MC and NC family-wise. In SMC, the true individual age at onset was known and subtracted from the actual age at sampling to generate the EYO variable. Hence, EYO has a negative value (<0) in the presymptomatic phase and a positive value (≥0) in the symptomatic phase.

#### Blood sample collection

Venous blood sampling was performed non-fasting at varying times of the day, using sodium heparin or EDTA as anticoagulant, before and after a change in protocol in 2015. Samples were centrifuged for 10 min at 2200*g* at +20°C within 60 min of sampling. The supernatant plasma was aliquoted into 1-ml polypropylene tubes and frozen at −80°C. Most samples were thawed on ice and realiquoted before refreezing and transportation to the laboratory in Gothenburg.

#### Simoa analysis of plasma NfL, T-tau, P-tau181 and GFAP

Plasma NfL, T-tau and GFAP were measured using the Quanterix Simoa^TM^ Human Neurology 4-plex A Assay (Quanterix Corporation). The lowest limit of quantification (LLOQ) and pooled coefficient of variation for this assay were 0.241 pg/ml and 12.0% for NfL, 0.467 pg/ml and 12.9% for GFAP and 0.053 pg/ml and 12.2% for T-tau. The P-tau181 assay was performed using the Simoa HD-1 instrument (Quanterix Corporation), with an LLOQ of 0.5 pg/ml and coefficients of variation <20%, as previously described.^14^

#### CSF collection and analysis

CSF samples were collected between 1993 and 2015. Immediately after collection into polypropylene tubes the CSF was centrifuged at 3000*g* at +4°C for 10 min. The supernatant was pipetted off, aliquoted into polypropylene cryotubes and stored at −80°C. Aβ and Tau peptide concentrations were analysed twice each: Aβ two times in 2016 and Tau in 2016 and in 2019. All analyses were performed at the Clinical Neurochemistry Laboratory at the Sahlgrenska University Hospital, Mölndal, Sweden by board-certified laboratory assistants, blind to clinical data.

CSF Aβ peptide concentrations were measured using electrochemiluminescence technology, with the MS6000 Human Abeta 3-Plex Ultra-Sensitive Kit (capture antibody 6E10), as recommended by the manufacturer (Meso Scale Discovery). CSF P-tau181 concentrations were measured by the INNOTEST® phospho-tau 181P enzyme-linked immunosorbent assay (ELISA; Fujirebio Europe)^15^ and T-tau by using a sandwich ELISA (INNOTEST TAU-Ag, Fujirebio Europe), designed to measure all tau isoforms regardless of phosphorylation status.^16,17^

#### *APOE* genotyping

The *APOE* genotyping was performed for single nucleotide polymorphisms (SNPs) rs7412 and rs429358 using TaqMan® SNP Genotyping Assays (ThermoFisher) according to the manufacturer’s protocol. The amplified products were run on 7500 fast Real-Time PCR Systems (ThermoFisher). Participants were annotated as *APOE* ɛ4-positive if carrying one or two alleles of ɛ4.

#### *APP* and *PSEN1* genotyping

Exons 16 and 17 in *APP* were resequenced to screen for the p.KM670/671NL^18^ and p.E693G^19^ mutations. Exon 6 was resequenced to detect the *PSEN1* p.H163Y mutation.^20^ AmpliTaq Gold® 360 PCR Master Mix (ThermoFisher) was used for DNA amplification. Primer sequences and PCR conditions are available upon request. Sanger sequencing was performed using BigDye™ Terminator v3.1 Cycle Sequencing Kit (ThermoFisher) in both forward and reverse directions and analysed using ABI3500 Genetic Analyzer (ThermoFisher).

#### Statistical analysis

##### Cross-sectional analyses

Descriptive statistical analyses were performed to compare groups of PMC, SMC and NC controls. Group comparisons were performed using either unpaired *t*-tests or Kruskal–Wallis and Mann–Whitney U-tests for normally distributed and skewed data, respectively. Descriptive statistics of categorical variables were performed using Fisher's exact test. Spearman correlations were calculated between plasma and CSF biomarker concentrations, based on cross-sectional data available on dates with matching plasma and CSF data. Also, Spearman correlations were calculated between plasma biomarker concentrations, age and EYO.

*P*-values < 0.05 were considered significant and always calculated from two-sided tests. Correction for multiple comparisons was done using FDR correction, with Q set to 5%.^21^

##### Longitudinal analyses

The effects of mutation status (MC or NC) and EYO on longitudinal plasma concentrations were assessed by using mixed-effects models. In the mixed-effects models, the fixed-effects predictors were defined as EYO, EYO^2^, mutation status and the interaction between mutation status and EYO (mutation status × EYO). ‘Individual’ was included as a random intercept to account for within-subject correlations. Robust estimators of variance were applied in all the models due to non-normal data and restricted maximum likelihood estimation was used. Additional sensitivity analyses were performed to exclude plasma biomarker data that were either extreme outliers (>3 × IQR) or when plasma samples had been processed using EDTA anticoagulant (in five PMC, six SMC and five NC samples). All results were adjusted for *APOE* ɛ4+ status (ɛ4 present or absent) and sex. The separation of 95% confidence bands for the longitudinal trajectories of MC and NC versus EYO was used to estimate when in time plasma biomarkers in the two groups started to diverge. The number of participants contributing to each sampling occasion in the longitudinal analyses is shown in Supplementary Table 4.

Statistical calculations were performed using SPSS 27.0 (IBM Corporation, Armonk, NY, USA) and R (R version 4.0.3, the R Foundation for Statistical Computing Platform), except for mixed-effects models that were done in STATA MP 15.1.

### Data availability

The data that support the findings of this study are not publicly available, in order to maintain the privacy of the research participants. The data are, however, available from the corresponding author upon reasonable request.

## Results

### Sample cohort and demographics

A total of 75 samples (24 PMC, 9 SMC and 42 NC) were included in cross-sectional analyses at baseline. The longitudinal analysis included 164 samples (87 MC and 77 NC) from the same 75 individuals (Supplementary Fig. 1 and Supplementary Table 1). The mean number of plasma sampling occasions per subject was 2.3 ± 1.6 (mean ± SD), with a range of 1–8, and the mean total follow-up time was 6.1 ± 7.5 (range of 0–23) years.

The demographics of the cohort are shown in Table 1, showing subgroups of PMC, SMC and NC as applied in the cross-sectional analyses. Furthermore, baseline characteristics of NC and MC groups as applied in the longitudinal analyses did not show statistical differences in age at baseline, EYO, proportions of *APOE* ɛ4+ status and sex (data not shown). The ages at baseline (mean ± SD) were normally distributed in the NC (47 ± 17) and MC (45 ± 11) groups. Principal component analysis did not show any effects of time of storage on plasma biomarker concentrations, neither did sex, choice of anticoagulant (EDTA/sodium heparin) or *APOE* ɛ4+ status (data not shown).

**Table 1:** Demographics

	PMC	SMC	NC
	*n* = 24	*n* = 9	*n* = 42
Age, years, median (range)	39 (27–53)	59 (55–66)*^,§^	43 (20–86)
EYO, years, median (range)	−15 (−26 to −2)	4 (1 to 10)*^,§^	−10 (−34 to 34)
Sex, female:male (%)	8:16 (33:67)	2:7 (22:78)	16:26 (38:62)
*APOE* ɛ4+, *n* (%)	12 (50)	2 (22)	18 (43)
Genotype, *n*			
ȃ*APPswe* p.KM670/671NL	10	3	
ȃ*APParc* p.E693G	7	6	
ȃ*PSEN1* p.H163Y	7	0	
CDR, median (range)	0 (0)	2 (0.5–3)	0 (0)
MMSE, median (range)	30 (27–30)	NA	29 (27–30)
Follow-up, years, mean ± SD	9 ± 9	1 ± 2	6 ± 7

Clinical characteristics at baseline and follow-up time. Two PMC converted (had symptom onset) during follow-up in the *APPswe*, four in the *APParc* and one in the *PSEN1* family. Reported *P*-values are unadjusted, but they all remained significant after correction for multiple testing. CDR = Clinical Dementia Rating scale; EYO = estimated years to expected onset (in family); MMSE = Mini-Mental State Examination test.

Significant *P*-value (*P* < 0.01) in comparison to NC.

Significant *P*-value (*P* < 0.001) in comparison to PMC.

### Plasma NfL, P-tau181, T-tau and GFAP

#### Cross-sectional analyses

**Figure 1:**
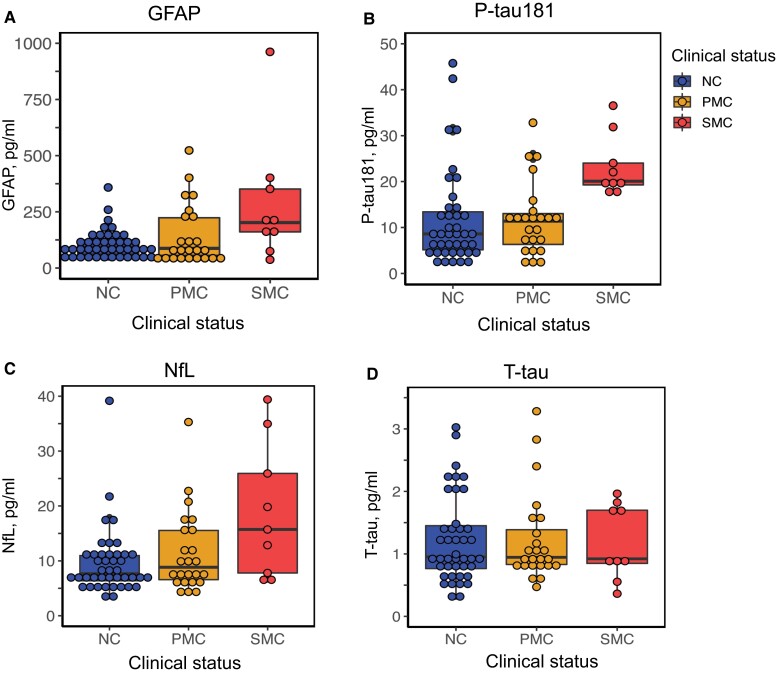
**Cross-sectional data, plasma biomarkers.** Cross-sectional plasma concentrations of (**A**) GFAP, (**B**) P-tau181, (**C**) NfL and (**D**) T-tau at baseline. Only P-tau181 was significantly increased in SMC compared to both NC (*P* < 0.001) and PMC (*P* < 0.01). Increases of NfL and GFAP in SMC compared to NC were not statistically significant (*P* = 0.057 and *P* = 0.058) as calculated with Kruskal–Wallis test.

At baseline, when analysing all families together, plasma P-tau181 levels were significantly higher in SMC compared to PMC (9 SMC, 24 PMC, Kruskal–Wallis *P* = 0.003) and NC (42 NC, Kruskal–Wallis *P* < 0.001; Fig. 1 and Table 2). The median concentrations of NfL and GFAP were doubled in SMC compared to NC, although these results did not reach statistical significance (Kruskal–Wallis, *P* = 0.057 and *P* = 0.058; Fig. 1 and Table 2). Exploratory subanalyses of the separate mutations at baseline are illustrated in Supplementary Figs 3–5.

**Table 2:** Plasma biomarker concentrations at baseline, cross-sectional data

	PMC	SMC	NC
	*n =* 24	*n =* 9	*n =* 42
P-tau181, pg/ml	11.4 (2.1–32.8)	20.1 (17.4–36.5)*^,§^	8.6 (1.7–45.8)
T-tau, pg/ml	0.9 (0.5–3.3)	0.9 (0.4–2.0)	1.0 (0.3–3.0)
NfL, pg/ml	8.8 (3.9–35.3)	15.7 (6.0–39.4)	7.7 (3.0–39.2)
GFAP, pg/ml	87.5 (28.7–523.7)	202.1 (36.7–961.9)	94.6 (32.7–358.1)

All biomarker values are expressed as median (range). Mann–Whitney U-test and Kruskal–Wallis test were used for significance testing. Differences in NfL and GFAP between SMC and NC only reached a trend (*P* = 0.057 and *P* = 0.058). Reported *P*-values are unadjusted, but they all remained significant after correction for multiple testing.

Significant *P*-value (*P* < 0.001) in comparison to NC.

Significant *P*-value (*P* < 0.01) in comparison to PMC.

#### Longitudinal analyses

Results from the statistical analyses of longitudinal sampling using mixed-effects models are shown in Supplementary Table 2. The mixed-effects models showed that concentrations of NfL, GFAP and P-tau181, but not T-tau, were increased in MC compared to NC. Similarly, NfL, GFAP and P-tau181, but not T-tau, increased with time as measured by fixed effects EYO and mutation status × EYO (Supplementary Table 2). In an exploratory analysis, the longitudinal biomarker data were stratified and analysed separately for each family (mutation) using the pooled NC as reference. The results suggest that individual mutations might have different effects on the plasma biomarker levels (Supplementary Fig. 6 and Supplementary Tables 5–7).

A sensitivity analysis was performed to evaluate the effect of extreme outliers and the possible impact on biomarker levels using different anticoagulants (EDTA and sodium heparin) at sampling. Here, exclusion of extreme outliers only or both extreme outliers and EDTA samples (five PMC, six SMC, five NC) showed unchanged results in longitudinal analysis, with higher GFAP (*P* < 0.001), NfL (*P* < 0.01) and P-tau181 (*P* < 0.001) levels in MC compared to NC. Fixed-effects EYO and mutation status × EYO were also not affected by exclusion of extreme outliers and EDTA samples (data not shown). Altogether, the presence of an ADAD mutation showed an effect on plasma NfL, GFAP and P-tau181 levels in the longitudinal analysis, which was not completely verified in the smaller baseline cohort. The individual trajectories of longitudinal plasma biomarker levels are shown in Supplementary Fig. 2.

**Figure 2:**
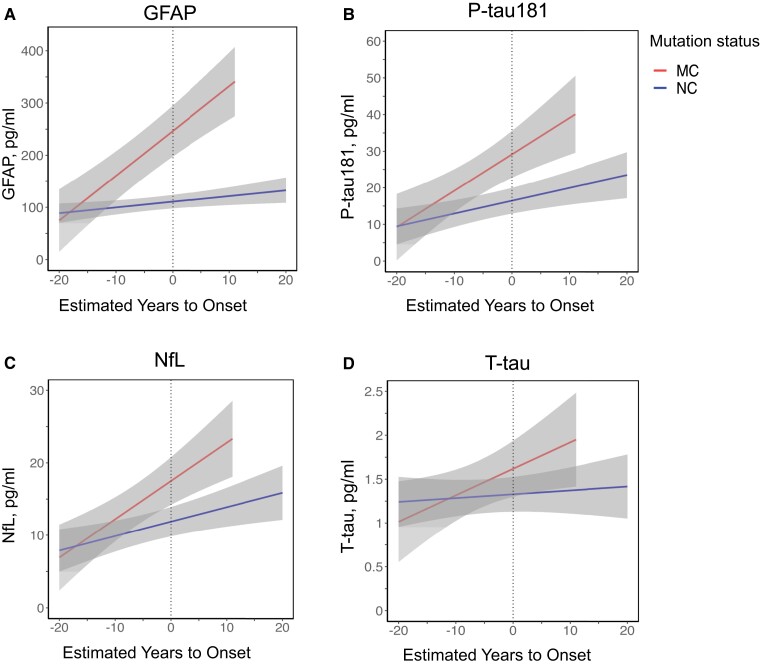
**Longitudinal data, plasma biomarkers.** Longitudinal plasma concentrations of (**A**) GFAP, (**B**) P-tau181, (**C**) NfL and (**D**) T-tau. Trajectories show the mixed-effects model fits with 95% confidence bands at the group level, separately for MC (*n* = 33, 87 samples) and NC (*n* = 42, 77 samples).

Plasma biomarker trajectories including 95% confidence bands in MC and NC showed that GFAP concentrations started to change first (approximately 10 years before expected onset), followed by P-tau181 (approximately 6 years before expected onset) and NfL (approximately 2 years before expected onset), as indicated by non-overlapping confidence bands (Fig. 2).

#### Correlations

CSF levels of Aβ peptides (Aβ38, Aβ40, Aβ42 and Aβ42/40 ratio), T-tau and P-tau181 were available from the same sampling dates in a subset of the plasma samples (*n* = 26–30) and used in correlation analyses with plasma Simoa biomarkers (Supplementary Table 3). No correlation was observed between plasma NfL or plasma T-tau and any of the CSF proteins. In contrast, plasma P-tau181 correlated to CSF T-tau (*r* = 0.602, unadjusted *P* < 0.001) and CSF P-tau181 (*r* = 0.563, unadjusted *P* < 0.01) also after FDR correction for multiple testing (24 comparisons). Plasma GFAP had an inverse correlation to CSF Aβ40 (*r* = −0.469 and unadjusted *P* = 0.016) that, however, was not significant after FDR correction (24 comparisons). The correlation between plasma P-tau181 and CSF T-tau and P-tau181 also remained significant after removing EDTA samples (*n* = 5) and extreme outliers (>3 × IQR; *n* = 1–3; Supplementary Table 3).

Plasma P-tau181 (*P* < 0.01), NfL (*P* < 0.001) and GFAP (*P* < 0.001) were positively correlated with age and EYO in MC (*n* = 33), but not in NC (*n* = 42), after FDR correction for multiple testing (eight comparisons). Plasma T-tau showed no correlation to EYO or age (data not shown).

## Discussion

In this explorative longitudinal study of plasma biomarkers in a Swedish ADAD cohort (*APPswe*, *APParc* and *PSEN1* p.H163Y), the major finding was that plasma NfL, P-tau181 and GFAP concentrations, but not T-tau, were increased in MC compared to controls. The first changes were detectable already in the preclinical phase, first by an increase in plasma GFAP approximately 10 years before onset, followed by P-tau181 and later NfL. This order of pathological changes as measured by plasma biomarkers is shown for the first time. Furthermore, plasma P-tau181 showed correlations with CSF levels of T-tau and P-tau181.

It remains unclear which CNS processes are measured by the astrocytic marker GFAP in plasma. GFAP has recently been suggested as a plasma biomarker of pathological accumulation of Aβ in brain and outperformed CSF GFAP in predicting the presence of cerebral Aβ using PET and abnormal Aβ levels in CSF.^22,23^ Furthermore, plasma GFAP previously outperformed plasma NfL and Aβ1-42/1-40 in predicting presence of brain Aβ using PET.^24^ Plasma GFAP increased already in preclinical sporadic Alzheimer's disease and showed similar discriminative accuracy as plasma P-tau181 and P-tau231 in predicting cerebral Aβ positivity using PET in healthy older adults.^25^ It has been speculated that Alzheimer's disease-associated cerebral amyloid angiopathy, including dysfunction of the blood–brain barrier and astrocytic activation in proximity of the microvasculature, might contribute to some of the discrepancy between CSF and plasma GFAP, but such mechanisms are not fully understood.^10^ Here, baseline plasma GFAP concentrations in symptomatic carriers were more than doubled compared to the levels in PMC and NC. Although the cross-sectional comparison did not reach significance, the longitudinal analysis did and showed a clear elevation of plasma GFAP in MC compared to NC, starting 10 years before the estimated age at symptom onset. The plasma GFAP levels increased over the lifespan in MC, as measured by EYO. This aligns with cross-sectional data from genetically determined Alzheimer's disease due to trisomy 21, which indicated that plasma GFAP has an inflection point approximately 10 years before onset of Alzheimer's disease in a Down syndrome cohort.^26^ Interestingly, in genetic frontotemporal dementia there was no presymptomatic increase in plasma GFAP and any possible increases in symptomatic phases might be mutation-specific,^27^ which gives further support for an Alzheimer's disease-specific increase of plasma GFAP in presymptomatic individuals. The correlation between plasma GFAP and CSF Aβ40 in our data was not statistically verified. Our subset with matching CSF was small and any true correlation needs to be assessed further in a larger cohort. This is the first report suggesting that plasma GFAP is one of the earliest blood-based biomarkers in ADAD to our knowledge. The timing could indicate that plasma GFAP could be a blood-based biomarker of early events such as glial activation and Aβ pathology, for use in both ADAD and sporadic Alzheimer's disease.

Longitudinal plasma P-tau181 data could separate SMC from NC with a high accuracy (area under the curve > 0.90) in a British cohort of ADAD.^28^ Our ADAD cohort is of comparable size and adds to the understanding of the role of P-tau181 in genetic forms of Alzheimer's disease. Here, the increase of plasma P-tau181 in SMC compared to NC was approximately 2.3-fold, which is analogous with previous reports in sporadic Alzheimer's disease and ADAD.^8,28,29^ Plasma P-tau181 was significantly higher in MC compared to NC approximately 6 years before estimated symptom onset. Such changes started 10 years earlier in the British ADAD cohort,^28^ indicating some variability in these trajectories of plasma P-tau181 also across ADAD cohorts of similar size. Finally, we show that plasma P-tau181 correlated to both CSF T-tau and P-tau181 in ADAD, consistent with other sporadic Alzheimer's disease cohorts.^7,14^ Altogether, our findings support the previous large amount of data that have highlighted plasma P-tau as an actual biomarker of Alzheimer's disease-related CNS tau pathology.

Plasma T-tau could not discriminate between MC and NC in this cohort and did not correlate to CSF biomarker concentrations, likely due to a peripheral production of tau or a rapid degradation in plasma, also supporting previous results of poor discriminative accuracy in sporadic Alzheimer's disease.^12,30^

Plasma and serum NfL were previously found to be increased in PMC in ADAD and NfL has previously been explored as an early biomarker of neurodegeneration in longitudinal ADAD cohorts.^31–34^ Blood-based NfL concentrations were further correlated to cognitive measures and cortical thinning^31,34^ as well as CSF NfL.^31^ Our longitudinal analysis showed somewhat weaker performance of plasma NfL than previous ADAD reports. Although plasma NfL increased in PMC compared to NC, this occurred much closer to onset in our dataset compared with the results from longitudinal data collected in the large Colombian *PSEN1* p.E280A ADAD cohort and the results from the international collaborative Dominantly Inherited Alzheimer Network (DIAN) cohort.^31–33^ Also, we found that plasma NfL did not correlate to CSF core Alzheimer's disease biomarkers. We hypothesize that this poor performance was caused by high intra-individual variability and the relatively limited number of CSF samples available for correlation analysis in our dataset.

Our longitudinal subanalyses of the three different families showed some variation in plasma biomarker levels between mutations and must be confirmed in a larger dataset before making firm conclusions. So far, previous ADAD data have indicated that plasma P-tau and serum NfL concentrations were not differentially affected by *APP*, *PSEN1* or *PSEN2* genetic groups.^28,31,33^

Development of blood-based surrogate biomarkers of neurodegeneration for clinical use has partly been delayed due to large variability and poor correlation to CSF biomarkers.^35–37^ Efforts have been made to develop guidelines for pre-analytical handling to improve reproducibility.^36,38^ Our results were based on a commercially available Simoa ultrasensitive biochemical assay method, with findings of large intra-individual variability and several extreme outliers. Such variability has been addressed in blood-based analysis of P-tau181 and NfL in a British ADAD setting.^28,33^ Although our follow-up time up to a 23-year period provides a very interesting cohort, the longitudinal analysis was limited by subjects who were lost from follow-up. The long follow-up time is also likely to introduce possible uncertainties about pre-analytical handling. Further, we allowed for both sodium heparin and EDTA anticoagulants and although the principal component analysis implied that this did not affect plasma biomarker concentrations at the group level, we found some outliers probably caused by matrix effects. Sensitivity analysis without EDTA samples and extreme outliers, however, indicated robust differences in plasma NfL, P-tau181 and GFAP concentrations in MC compared to NC. Storage effects over time did not seem to be an issue in our analysis, but participants were non-fasting and the varying time of sampling is likely to have introduced diurnal effects. We suggest that the intra-individual variability seen in both MC and NC here is attributable in part to pre-analytical handling issues, but true biological variations can also play a role. Last, other factors such as body mass index, kidney and liver function might influence plasma biomarker concentrations. Information of such clinical data was incomplete and not accounted for, which is a limitation of the study. The total variability in several biomarker concentrations remains an obstacle for statistical analysis and usefulness in clinical practice and trials, which emphasizes the importance of standardized operational procedures.

Our results have several very important implications. We suggest that plasma P-tau181, GFAP and NfL are feasible biomarkers to detect different Alzheimer's disease-related pathologies already in presymptomatic individuals. Furthermore, we show that plasma GFAP, recently being added to the collection of emerging blood-based biomarkers, is an early biomarker that appears to start to change before P-tau181 and NfL. These are the first published data on plasma GFAP in ADAD. Although the cohort is small, and we could not statistically determine a correlation to CSF Aβ levels, the timing of plasma GFAP changes observed aligns with the existing evidence in sporadic Alzheimer's disease. Thus, our results suggest that plasma GFAP is more strongly correlated to the early pathological accumulation of Aβ in CNS rather than to the downstream pathological accumulation of tau. If validated, these plasma biomarkers bring the potential to reflect the core events of CNS Alzheimer's disease pathology, such as amyloid pathology or glial activation (plasma GFAP) followed by accumulation of tangles (plasma P-tau) and later evidence of neurodegeneration (plasma NfL; A/T/N).^2,5,39^ These results remain to be replicated in larger cohorts and further studies are needed to investigate whether plasma GFAP also correlates to or can predict astrocytic activation.

## Supplementary Material

awac399_Supplementary_Data
