# Supplementary material for: Plasma biomarker profiles in autosomal dominant Alzheimer’s disease
Source: Brain. 2023 Jan 11;146(3):1132–40. doi: 10.1093/brain/awac399 (PMC9976964; doi:10.1093/brain/awac399)
Supplement: awac399_Supplementary_Data [file awac399_supplementary_data.pdf]

Supplementary Material

Johansson C, Thordardottir S, Laffita J, *et al.* Plasma biomarkers profiles in autosomal dominant Alzheimer Disease

Corresponding author: Caroline Graff, Karolinska Institutet, Dept NVS, Division of Neurogeriatrics, Center for Alzheimer Disease Research, Bioclinicum J10:20, Visionsgatan 4, 171 64 Solna, Sweden.
Tel: +46 (0) 73 383 93 99, **E-mail**: caroline.graff@ki.se

Table of content

Supplementary Tables

Supplementary Table 1. MC samples by mutation

Supplementary Table 2. Results from the longitudinal analyses of plasma biomarkers in all MC vs NC using mixed effects models

Supplementary Table 3. Correlation table, plasma and CSF biomarkers

Supplementary Table 4. Sampling occasions included in longitudinal analysis

Supplementary Table 5. Exploratory results from the longitudinal analyses of plasma biomarkers in *APPswe* MC vs NC using mixed effects models

Supplementary Table 6. Exploratory results from the longitudinal analyses of plasma biomarkers in *APParc* MC vs NC using mixed effects models

Supplementary Table 7. Exploratory results from the longitudinal analyses of plasma biomarkers in *PSEN1* MC vs NC using mixed effects models

Supplementary Figures

Supplementary Fig. 1. Flow chart of sample cohort

Supplementary Fig. 2. Exploratory longitudinal data, individual trajectories.

Supplementary Fig. 3. Exploratory cross-sectional data, *APPswe*

Supplementary Fig. 4. Exploratory cross-sectional data, *APParc*

Supplementary Fig. 5. Exploratory cross-sectional data, *PSEN1* p.H163Y

Supplementary Fig. 6. Exploratory analysis of longitudinal plasma biomarker levels, by mutation

Supplementary Table 1. MC samples by mutation

	PMC	SMC
	Samples	
APParc	16	19
APPswe	17	6
*PSEN1 p.*H163Y	26	3
Total	59	28

Samples analyzed in the longitudinal study, family-wise. Non-carriers from the three families were included together as the reference group in all analyses (n=42, 77 samples). In total 75 individuals and 164 samples were included.

Supplementary Table 2. Results from the longitudinal analyses of plasma biomarkers in all MC vs NC using mixed effects models

	EYO	EYO^2	Mut status	Mut status*EYO
	Estimate [SE]	Estimate [SE]	Estimate [SE]	Estimate [SE]
Plasma T-tau	ns	ns	ns	ns
Plasma P-tau181	0.339 [0.112]**	ns	13.401 [4.385]**	0.727 [0.270]**
Plasma NfL	0.201 [0.101]*	ns	5.014 [2.324]*	0.328 [0.140]*
Plasma GFAP	1.074 [0.490]*	ns	135.104 [32.813]***	7.931 [2.072]***

164 samples from all families were included, 87 samples from 33 MC and 77 samples from 42 NC. Table shows estimates, the standard error [SE] and statistical significance (*p<0,05, **p<0,01, ***p<0,001). All models were corrected for *APOE* status and sex. All results of a positive mutation status and mutation status*EYO effect remained significant in a sensitivity model removing EDTA samples (n=16) and outliers (>3*interquartile range). Mut status= Mutation status, here mutation carriers compared to non-carriers.

Supplementary Table 3. Correlation table, plasma and cerebrospinal fluid biomarkers

	CSF Aβ1-38 (n=26)	CSF Aβ1-40 (n=26)	CSF Aβ1-42 (n=26)	CSF Aβ1-42/1-40 (n=26)	CSF T-tau (n=30)	CSF P-tau181 (n=29)
Plasma T-tau	ns	ns	ns	ns	ns	ns
Plasma P-tau181	ns	ns	ns	ns	**0.602*** **	**0.563** **
Plasma NfL	ns	ns	ns	ns	ns	ns
Plasma GFAP	ns	-0.469*	ns	ns	ns	ns

Table of Spearman Rho correlation coefficients and significance levels (*p<0,05, **p<0,01, ***p<0,001). Reported significance levels are unadjusted, but P-values were corrected for multiple comparison (n=24) by FDR correction and annotated in bold if still significant. When removing EDTA samples (n=5) and outliers (>3*IQR) (n=1-3), correlation was unchanged between plasma P-tau181 and CSF biomarkers. ns= non-significant, p>0.05.

Supplementary Table 4. Sampling occasions included in longitudinal analysis

	Baseline	Sampling 2	Sampling 3	Sampling 4	Sampling 5	Sampling 6	Sampling 7	Sampling 8	Total
MC (n)	33	20	12	8	7	4	2	1	87
NC (n)	42	23	8	3	1				77
All (n)	75	43	20	11	8	4	2	1	164

Table indicating number of individuals contributing samples for the longitudinal biomarker analysis, separated by mutation status.

Supplementary Table 5. Exploratory results from the longitudinal analyses of plasma biomarkers in *APPswe* MC vs NC using mixed effects models

	EYO	Mut status	Mut status*EYO
	Estimate [SE]	Estimate [SE]	Estimate [SE]
Plasma T-tau	ns	ns	ns
Plasma P-tau181	1.141 [0.268]***	13.555 [4.470]**	0.793 [0.289]**
Plasma NfL	0.644 [0.168]***	7.571 [2.752]**	0.448 [0.181]*
Plasma GFAP	11.338 [1.702]***	253.189 [32.187]***	10.550 [1.852]***

Mixed effects models of longitudinal data. 100 samples were included (23 samples from 13 *APPswe* MC and 77 samples from 42 NC). Table shows estimates, the standard error [SE] and statistical significance (*p<0,05, **p<0,01, ***p<0,001). All models were corrected for *APOE* status and sex. Mut status= Mutation status, here *APPswe* mutation carriers compared to non-carriers.

Supplementary Table 6. Exploratory results from the longitudinal analyses of plasma biomarkers in *APParc* MC vs NC using mixed effects models

	EYO	Mut status	Mut status*EYO
	Estimate [SE]	Estimate [SE]	Estimate [SE]
Plasma T-tau	ns	ns	ns
Plasma P-tau181	ns	11.101 [5.136]*	ns
Plasma NfL	ns	6.077 [2.632]*	ns
Plasma GFAP	6.610 [1.862]***	75.381 [16.920]***	5.559 [1.965]**

Mixed effects models of longitudinal data. 112 samples were included (35 samples from 13 *APParc* MC and 77 samples from 42 NC). Table shows estimates, the standard error [SE] and statistical significance (*p<0,05, **p<0,01, ***p<0,001). All models were corrected for *APOE* status and sex. Mut status= Mutation status, here *APParc* mutation carriers compared to non-carriers.

Supplementary Table 7. Exploratory results from the longitudinal analyses of plasma biomarkers in *PSEN1* MC vs NC using mixed effects models

	EYO	Mut status	Mut status*EYO
	Estimate [SE]	Estimate [SE]	Estimate [SE]
Plasma T-tau	ns	ns	ns
Plasma P-tau181	0.383 [0.116]**	17.290 [5.164]**	0.985 [0.307]**
Plasma NfL	0.207 [0.062]**	ns	ns
Plasma GFAP	1.037 [0.476]*	55.878 [19.570]**	4.493 [1.332]**

Mixed effects models of longitudinal data. 106 samples were included (29 samples from 7 *PSEN1* MC and 77 samples from 42 NC). Table shows estimates, the standard error [SE] and statistical significance (*p<0,05, **p<0,01, ***p<0,001). All models were corrected for *APOE* status and sex. Mut status= Mutation status, here *PSEN1* mutation carriers compared to non-carriers.

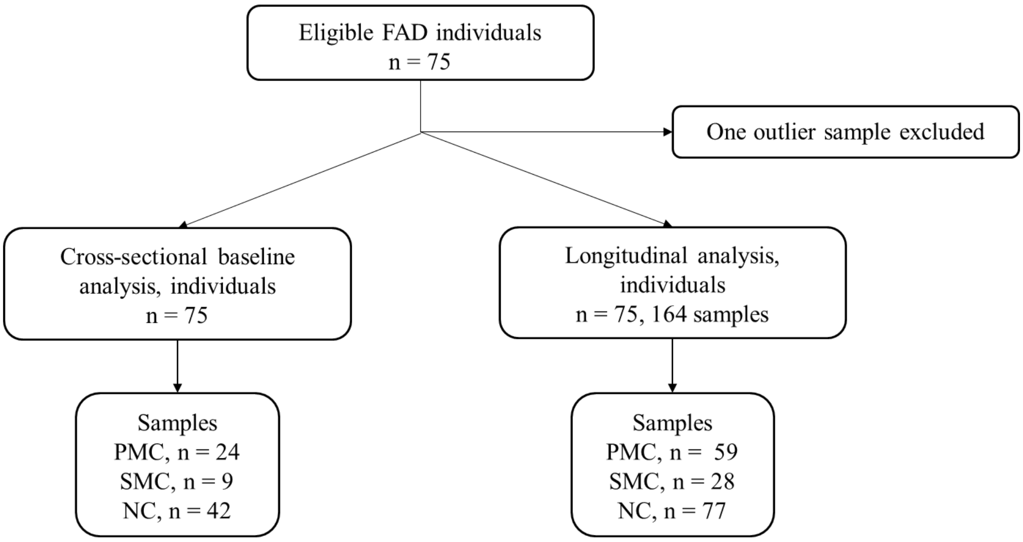

Supplementary Figure 1. Flow chart of sample cohort. 75 participants were eligible for inclusion in the plasma biomarker analyses. One NC outlier sample was removed from all analyses due to quality control issues. PMC= Presymptomatic mutation carriers, SMC= Symptomatic mutation carriers, NC= Non-carriers.

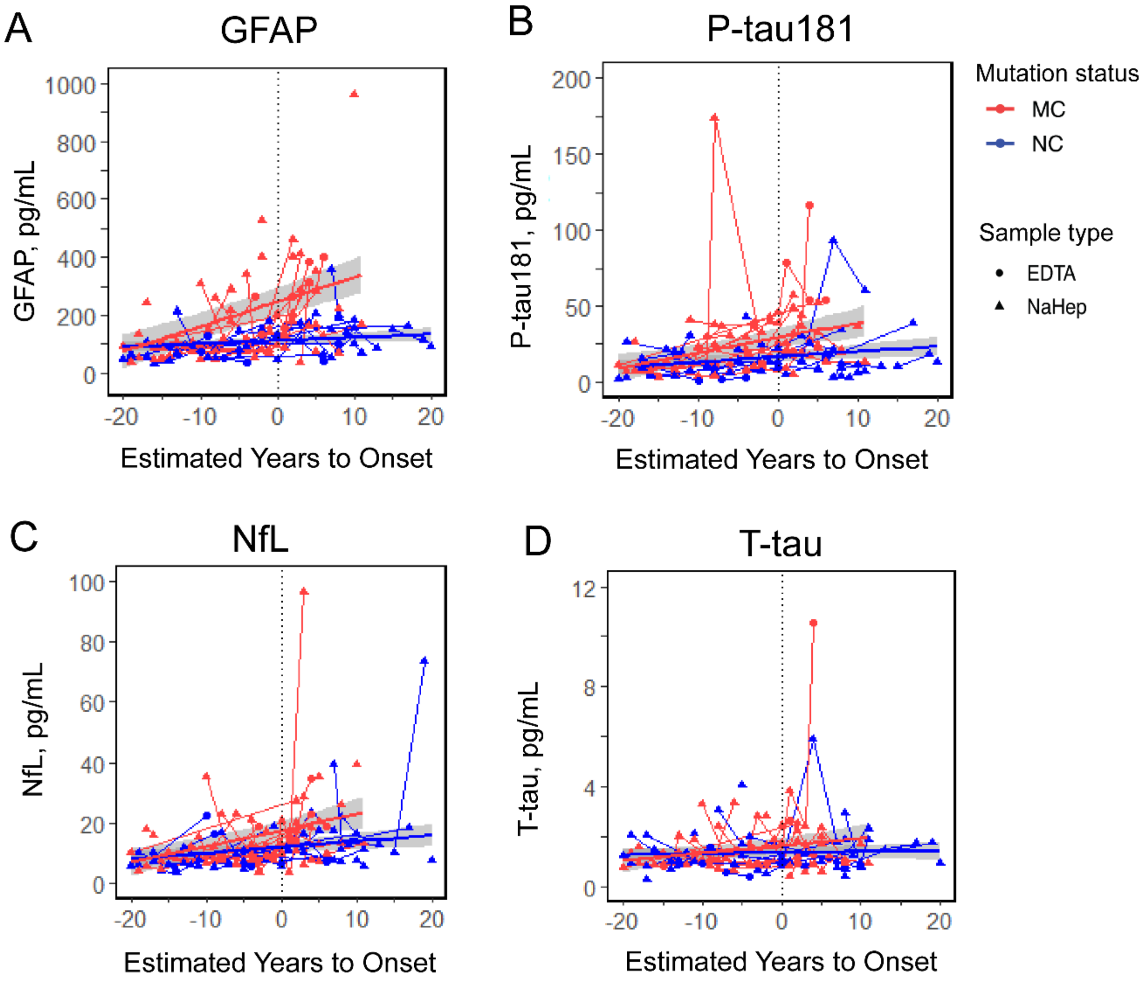

Supplementary Figure 2. Exploratory longitudinal data, individual trajectories. Longitudinal plasma concentrations of **(A)** GFAP, **(B)** P-tau181, **(C)** NfL and **(D)** T-tau. Trajectories show repeated measures at the individual level and mixed-effects model fits with 95% confidence bands at the group level in grey, separately for MC (n=33, 87 samples) and NC (n=42, 77 samples). Two presymptomatic MC in the *APPswe* family converted (had symptom onset) during follow-up, four in *APParc* and one in the *PSEN1* p.H163Y family. All sampling occasions with positive estimated years to onset (≥0) were in symptomatic individuals, except for one *PSEN1* MC with reduced penetrance. EDTA samples (n=16) and outliers (>3*interquartile range) were removed as part of a mixed models sensitivity analysis as described in text. NC = non-carriers, MC = mutation carriers. EDTA = ethylenediamine tetraacetic acid anticoagulant, NaHep = sodium heparin anticoagulant.

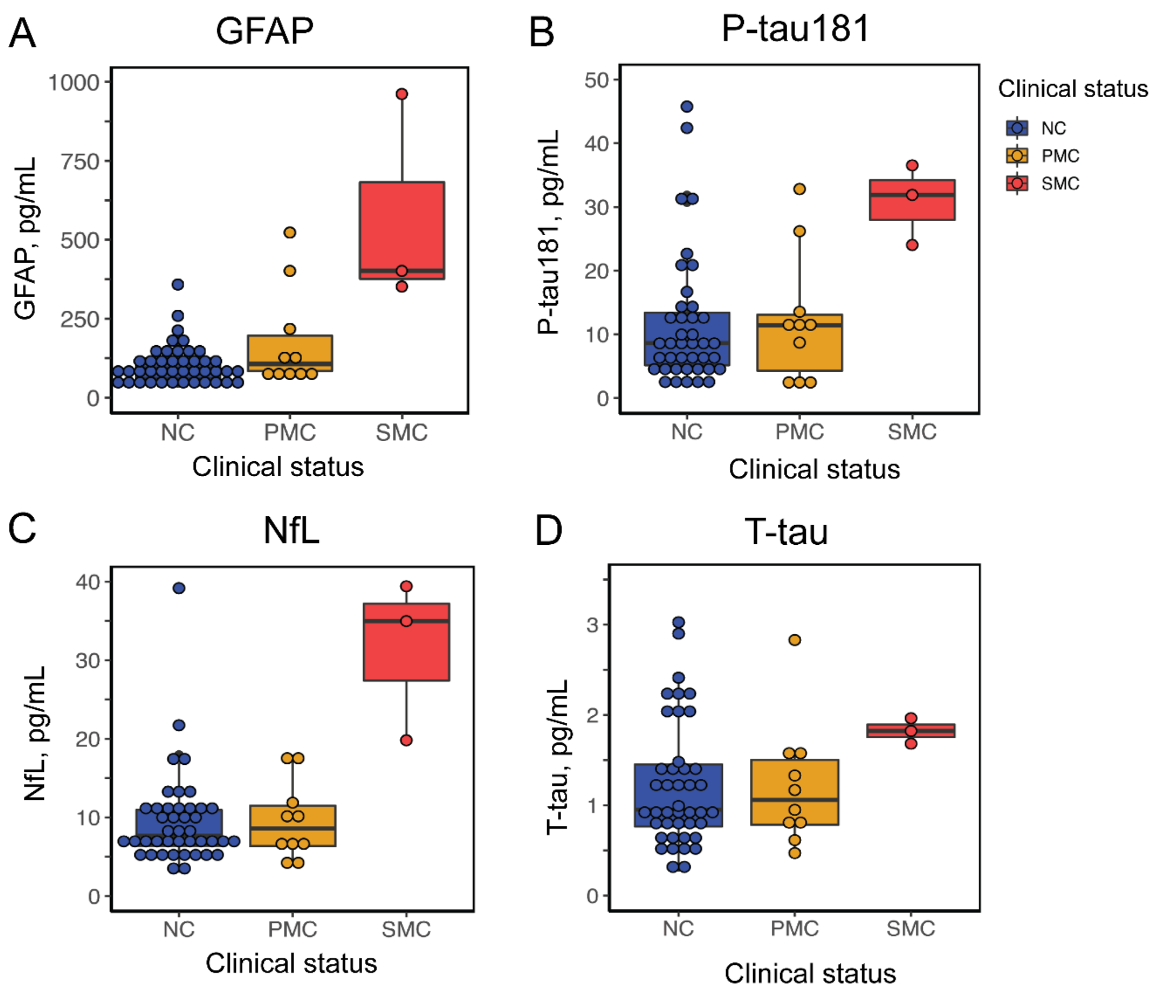

Supplementary Figure 3. Exploratory cross-sectional data, *APPswe*. Baseline data, plasma biomarkers in the *APPswe* family. NfL was significantly increased in SMC compared to both NC and PMC ($p<0.05$). GFAP and P-tau181 was increased in SMC compared to NC, but not PMC ($p<0.05$), as calculated with Kruskal Wallis test.

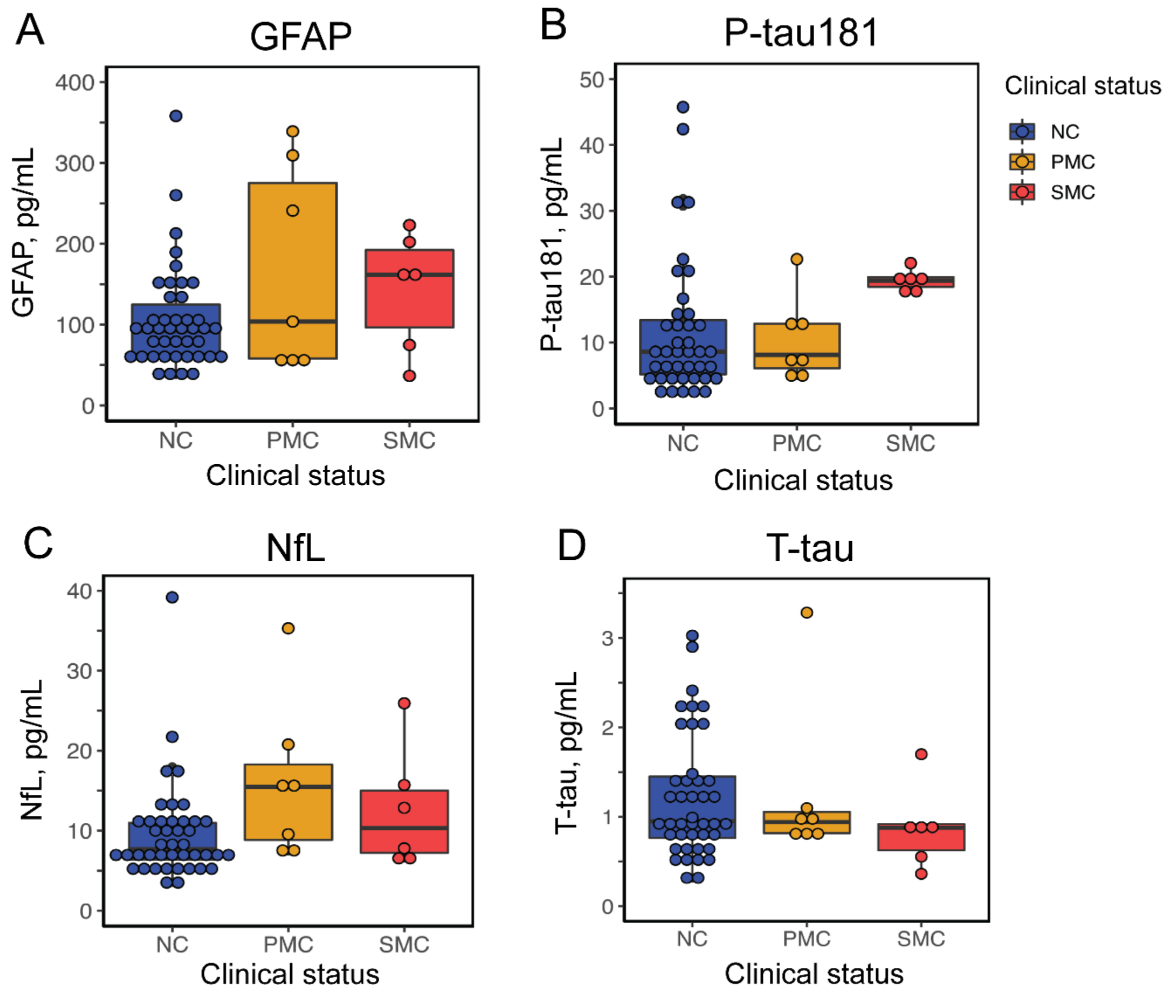

Supplementary Figure 4. Exploratory cross-sectional data, *APParc*. Baseline data, plasma biomarkers in the *APParc* family. P-tau181 was significantly increased in SMC compared to NC ($p<0.05$), but not PMC, as calculated with Kruskal Wallis test. No other between-group differences were detected.

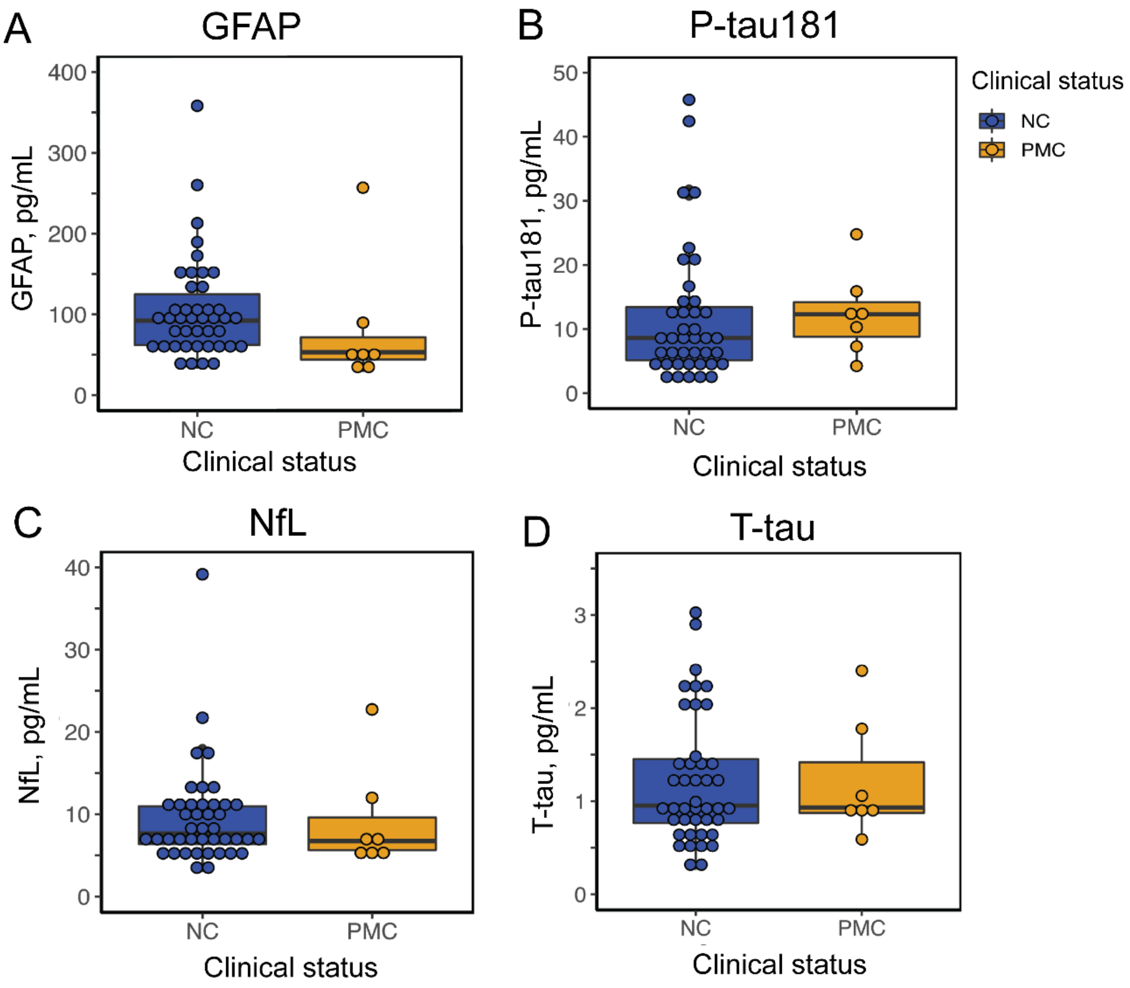

Supplementary Figure 5. Exploratory cross-sectional data, *PSEN1* p.H163Y. Baseline data, plasma biomarkers in the *PSEN1* p.H163Y family. GFAP was lower in PMC compared to NC ($p<0.05$) as calculated with Mann-Whitney U test. No other between-group differences were detected. This finding is likely explained by the younger age of *PSEN1* PMC (median age 31, range 27-44) compared to NC (median age 43, range 20-86).

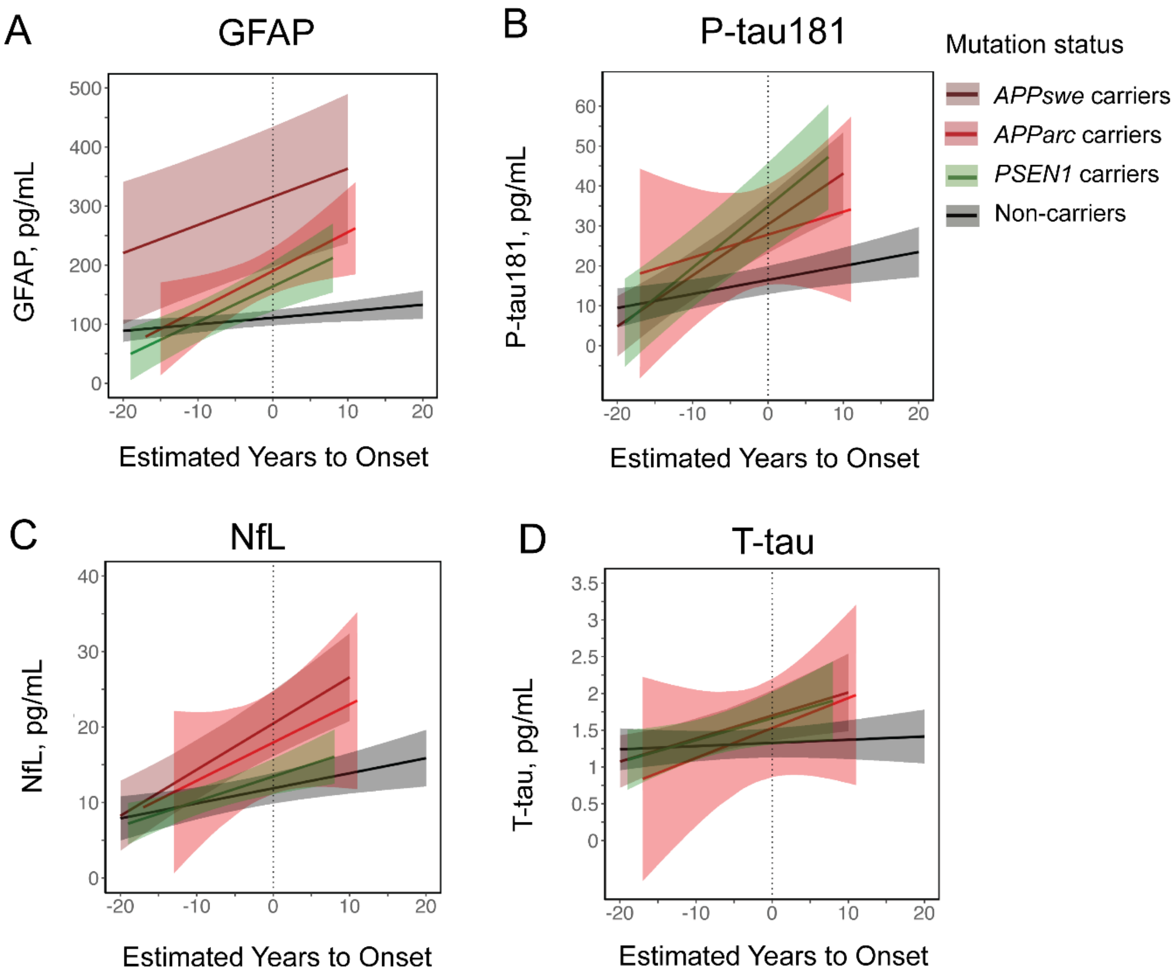

Supplementary Figure 6. Exploratory analysis of longitudinal plasma biomarker levels, by mutation. Longitudinal plasma concentrations of **(A)** GFAP, **(B)** P-tau181, **(C)** NfL and **(D)** T-tau. Trajectories show mixed-effects model fits with 95% confidence bands at the group level, separately for *APPswe* MC (n=13, 23 samples), *APParc* MC (n=13, 35 samples), *PSEN1* p.H163Y MC (n=7, 29 samples) and NC (n=42, 77 samples). NC = non-carriers, MC = mutation carriers.
